# Toward New Directions in Human Biology: A Roadmap for Anthropological Causal Inference With Observational Data

**DOI:** 10.1002/ajhb.70149

**Published:** 2025-09-22

**Authors:** Elijah J. Watson, Delaney J. Glass, Lucia C. Petito

**Affiliations:** ^1^ Department of Anthropology Northwestern University Evanston Illinois USA; ^2^ Department of Anthropology University of Toronto – St. George Toronto Ontario Canada; ^3^ Department of Preventive Medicine Northwestern University Feinberg School of Medicine Chicago Illinois USA

**Keywords:** causal inference, confounding, directed acyclic graphs, observational data analysis, statistical analysis

## Abstract

Human biologists seek to understand how cultural, environmental, and biological forces shape observed patterns of human variation. Yet contemporary insights and approaches to observational causal inference remain underutilized in the field. We outline a structured but flexible roadmap for causal inference in human biology that begins with theory development, defines causal questions and estimands, employs directed acyclic graphs (DAGs) to clarify assumptions, and evaluates key identification criteria prior to statistical analysis. We position this framework within a spectrum of causal inference traditions, spanning from interventionist approaches rooted in well‐defined, manipulable exposures to realized approaches that engage historically situated and ecologically embedded phenomena. Rather than offering a prescriptive checklist, we frame this toolkit as an opening: a step toward anthropological causal inference that integrates transparency, theoretical and methodological coherence, and the epistemological commitments of the biocultural synthesis in human biology and anthropology.

## 1 Introduction

Observational data are the bread and butter of human biology, but human biology has lagged behind allied fields that have adopted contemporary frameworks for observational causal inference. Even when interpretations of statistical associations are couched in language that carefully avoids the word “causal,” we assert that causal inference is the implicit goal in human biology whenever confounder‐adjusted associations are estimated (Hernán 2018; Hernán et al. 2002). Why? Researchers adjust for confounders to get closer to causal understandings of human biology from complex, real‐world data. If that is not causal inference, then what is?

The phrases “correlation does not imply causation” and “all models are wrong, but some are useful” are well known (Box and Draper 1987; Pearl 2021). However, neither maxim provides a roadmap for determining which correlations imply causation and how to design studies and statistical models to make causal inferences. In a paper addressing biological anthropologists, Smith (2019) wrote, “the issues with using observational data for causal inference include the importance of a theoretical background for identifying confounding effects, the difficulties in transferring theoretical concepts into practical measurements, and the limitations of research designs for controlling sampling biases. These issues precede the application of statistical technologies.” If we envision a general roadmap to causal inference, statistical estimation and interpretation are final steps following the prerequisite steps of specifying a causal research question and assessing *identifiability*, or whether we can estimate the causal parameter from the data we have collected (McElreath 2020; Petersen and van der Laan 2014).

Human biologists have drawn attention to issues of statistical inference in our field and biological anthropology, particularly concerning the misinterpretation of *p*‐values (Valeggia and Fernández‐Duque 2022). Relatedly, a recent toolkit article in this journal highlighted opportunities and considerations for using secondary datasets to answer questions in human biology (Rosinger and Ice 2019). However, there has been limited focus on the translation and dissemination of formal causal inference frameworks, which have become widely adopted in a growing number of fields, including epidemiology (Glymour 2006; Hernán and Robins 2020; Petersen and van der Laan 2014), sociology (Morgan and Winship 2007), psychology (Rohrer 2018, 2024), and economics (Angrist and Pischke 2009; Heckman 2005).

In human biology specifically, however, formal causal inference remains underutilized. While allied fields have increasingly adopted frameworks grounded in counterfactual reasoning, graphical models, and identification logic (Glymour 2006; Hernán and Robins 2020; Morgan and Winship 2007; Rohrer 2018), our field lacks a roadmap tailored to its empirical realities and epistemological goals. Drawing inspiration from recent guides in sociology (Lundberg et al. 2021), evolutionary human sciences (Bulbulia 2024a, 2024b, 2024c, 2024d; Major‐Smith 2023), ecology and behavior (Laubach et al. 2021), and epidemiology (Dang et al. 2023; Petersen and van der Laan 2014), this paper offers a comparable roadmap tailored for human biology.

Our approach is motivated by causal inference developments in social epidemiology and sociology, particularly *realized causal inference* (Schwartz and Prins 2025), which emphasizes retrospective causal explanation in historically and socially situated contexts. We focus on confounder adjustment strategies because they align with a common type of data structure in human biology: long‐term, field‐based studies rich in contextual data, but not easily amenable to the kinds of exogenous variation that enable quasi‐ or natural experimental approaches. However, we briefly introduce quasi‐experimental designs in the discussion section and encourage human biologists to become conversant in these methods. Doing so will strengthen our ability to critically engage with and build upon quasi‐experimental research relevant to human biology, such as the growing literature in economics and demography that leverages natural experiments to study the fetal and early life origins of human growth, health, and development (Almond and Currie 2011; Torche and Nobles 2024).

What follows is not a checklist or a rigid framework, but a flexible and integrative guide. It is grounded in practical tools and the philosophy of science, designed to help human biologists clarify their causal questions, evaluate the assumptions needed to answer them, and reason responsibly when those assumptions are only partially met. In this paper, we provide a broad introduction to the general fundamentals of a scientific workflow for human biology that integrates causal inference with observational data. For readers seeking complementary perspectives and deeper technical elaboration, we encourage engagement with the recent special issue on causal inference in *Evolutionary Human Sciences*, particularly the four‐part series by Bulbulia (2024a, 2024b, 2024c, 2024d), which offers an accessible and rigorous walkthrough of causal diagrams, confounding, mediation, measurement error, and study design.

Our goal is to bring human biology into deeper conversation with methodological advances in causal inference without losing sight of anthropological and evolutionary theory, not by importing other fields' strategies wholesale, but by adapting and developing approaches that align with our epistemological, ethical, and empirical commitments. In doing so, we sketch a vision of what anthropological causal inference in human biology might look like: rigorous but pragmatic and transparent but situated.

### 1.1 Modes of Quantitative Inquiry: Prediction, Description, and Causal Inference

Quantitative studies typically pursue one of three aims: prediction, description, or causal inference (Hernán et al. 2019). Predictive studies aim to forecast outcomes based on observed variables, such as using birth weight and other characteristics to predict inflammation in adulthood, without necessarily explaining why those patterns arise. True predictive modeling is a distinct statistical task from description: it involves training a model on existing data and evaluating its performance on new or held‐out data to optimize accuracy, often via cross‐validation (Hastie et al. 2009; James et al. 2013).

Descriptive studies aim to quantitatively summarize features in the real world, while causal inference seeks to summarize features of counterfactual worlds. For example, descriptive studies may estimate the prevalence of a phenotype or environmental characteristic, or examine how such features vary across population subgroups (Fox et al. 2022; Gerring 2012; Lesko et al. 2022).

While coefficients from regression analyses that adjust for multiple putative confounders are sometimes described as “descriptive” or “predictive” associations, we encourage human biologists to reserve the term *descriptive* for analyses that aim to reflect the real world rather than a counterfactual one, and *predictive* for analyses that aim to build models for making out‐of‐sample predictions (Arif and MacNeil 2022; Hernán 2018; Hernán et al. 2019). Adjustment for a single or small number of variables (e.g., age‐ or sex‐standardization) often remains appropriate in descriptive work to support “fair” comparisons across groups, but this requires careful reflection on what counts as fair or unfair adjustment (see Duan et al. 2008; Kaufman 2017). Within a population, if the goal is to describe how a feature varies across covariates, researchers could report stratified descriptive estimates instead of coefficients from a multiple regression model; for example, reporting trait prevalences stratified by sex, age, or both (Lesko et al. 2022).

Causal inference, in contrast, asks what outcome would have been observed if an exposure had been different. It is this third mode of quantitative inquiry that motivates the framework developed in the remainder of this paper.

### 1.2 The Fundamental Problem of Causal Inference

Suppose we want to know whether being born with low birth weight affects inflammation in adulthood. For any given individual, we can only observe the outcome under the condition they actually experienced (either low birth weight or not). We cannot observe the counterfactual outcome: what would have happened if their birth weight had been different. This reflects the fundamental problem of causal inference: we cannot directly observe both potential outcomes for the same person (Holland 1986).

Because of this, causal effects must be inferred, not observed. While we cannot estimate individual‐level effects, we can estimate average causal effects across groups, provided we can assume that the groups differ only in terms of their exposure (i.e., that there is no confounding). This logic underpins the design of randomized controlled trials (RCTs), in which randomization aims to balance both observed and unobserved factors across treatment groups, allowing for valid group‐level comparisons. In the next section, we examine how randomized experiments—and attempts to emulate them using observational data—have shaped the logic of causal inference more broadly, and where their limits begin to show.

### 1.3 Randomized Controlled Trials and Interventional Causal Effects

Randomized controlled trials are widely regarded as a gold standard for causal inference because, in principle, randomization eliminates confounding. They are also built around “well‐defined interventions,” enabling researchers to estimate what would happen under a specific manipulation.

The logic of the RCT has deeply shaped observational causal inference, particularly in public health and medicine. One influential framework is target trial emulation, which aims to improve observational comparative effectiveness studies by modeling them after the structure of a hypothetical RCT (Hernán et al. 2022). This includes identifying a clinical decision where multiple well‐defined interventions exist (e.g., choice of anti‐hypertensive medications), identifying eligibility criteria, specifying a start time and causal contrast, and applying appropriate adjustment strategies. Target trial emulation has been especially useful in pharmacoepidemiology and health policy evaluation, where interventions can be clearly specified in principle, even if ethics, resource constraints, or finances make randomized studies not feasible.

Interventional logic is particularly useful when the goal is to inform policy or evaluate treatments. It is forward‐looking, concerned with what will happen if we do X, rather than retrospective, which asks what caused Y. As Gelman and Imbens (2013) note, this distinction maps onto the difference between estimating the “effects of causes” versus the “causes of effects.” While intervention‐focused causal inference can sharpen interpretation in settings where hypothetical interventions are well‐defined, it is often ill‐suited to the explanatory, situated questions that animate human biology. Exposures commonly studied in human biology, such as nutrition, stress, or anthropometric traits, are historically embedded, socially patterned, and ecologically contingent. There is rarely a single, well‐defined intervention that corresponds to a comparison across higher or lower values of these measures. For instance, differences in anthropometric status might reflect prenatal nutrition, childhood illness, chronic food insecurity, or intergenerational social exclusion, each with distinct mechanisms and no single or uniform interventional analogue.

Even in more traditionally intervention‐focused domains like epidemiology, many interventional observational causal inference studies fall short of clearly specifying a well‐defined intervention. Eisenberg‐Guyot et al. (2025) reviewed 146 epidemiologic studies using causal inference methods with observational data and identified 29 that aimed to estimate the effects of hypothetical interventions. Of those, only 3% (1 study) clearly described how the exposure would actually be intervened upon. This suggests that even when the ideal of a well‐defined intervention is only partially met, epidemiologists commonly pragmatically apply causal inference tools to reduce avoidable bias and strengthen interpretability.

Randomized controlled trials and efforts to emulate them with observational data have contributed substantially to our understanding of human physiology, development, and health. They offer a valuable set of principles for clarifying assumptions and strengthening causal claims. Yet not all human biological questions lend themselves to tightly defined interventions or trial‐based thinking. Much of the field of human biology is grounded in anthropologically informed research that aims to describe and explain the patterned distribution of biological traits rather than predict outcomes under hypothetical interventions. Advancing causal reasoning in this context calls for approaches that accommodate these aims. Disciplines such as social epidemiology, which wrestle with similar challenges, have developed complementary frameworks that expand both the range of causal questions we can ask and the forms of evidence we can use to answer them.

### 1.4 Realized Causal Inference and the Biocultural Synthesis

While clinical medicine requires efficient and precise knowledge of how and why specific treatments work, an anthropological approach to human biology aims to more broadly understand how and why human biological variation arises in the first place. Understanding and explaining biological variation therefore demands deep engagement with the particulars of specific, situated cases, alongside iterative and comparative syntheses across related and unrelated cases, whether spanning ecologies, time periods, populations, or phylogenies (Nunn 2011).

Causal inference provides tools for explaining variation by comparing what happened to what might have happened under different conditions. *Realized causal inference*, developed in social epidemiology by Schwartz et al. (2016, 2017) and further developed in *Causal Inference and the People's Health* (Schwartz and Prins 2025), places particular emphasis on how exposures unfold in specific historical, social, and biological contexts. While sharing similar counterfactual logic with interventionist approaches, realized causal inference is suited to observational settings where interventions may be infeasible or ill‐defined. As Schwartz et al. (2016) argue, conventional interventionist frameworks may inadvertently sideline structurally rooted phenomena that resist neat operationalization such as racism, inequality, or capitalism because interventionist paradigms privilege well‐defined manipulable exposures. Realized causal inference expands the scope of causal analysis to include such forces, not by rejecting counterfactual reasoning, but by situating it within the lived realities and embedded histories that shape exposure–outcome relationships.

The orientation of realized causal inference resonates with Levins' (1966) model‐building philosophy for population biology, which holds that scientific models must often trade precision for realism and generality. While interventional approaches, idealized through the randomized controlled trial, gain precision through tightly defined exposures, they may sacrifice external validity when trial conditions fail to reflect broader or more heterogeneous realities. Realized causal inference, by contrast, trades some degree of (interventional) precision for context‐sensitive realism by allowing exposures that are not well‐defined or manipulable in an experimental sense to be examined as causes.

Although findings from a single study may not always be broadly generalizable, realized causal inference can still contribute to generalizable knowledge through *inference to the best explanation* (IBE). First articulated by Harman (1965), IBE offers a philosophical and practical strategy for reasoning under uncertainty. It asks: What is the most plausible explanation for the observed pattern, given competing theories and available knowledge? In social science, IBE often unfolds in three stages: researchers observe a pattern and generate plausible explanations; they then test the implications of those explanations using additional data; and finally, they evaluate which explanation best fits the full body of evidence (Spirling and Stewart 2024).

As Krieger and Davey Smith (2016) argue in epidemiology, IBE is particularly useful when exposures cannot be ethically or practically randomized, and when the goal is to reconstruct causal histories shaped by social, political, and historical forces. In such cases, bias is rarely eliminated, but perfection is not the goal. What matters is epistemic transparency and the systematic comparison of plausible explanations. By clarifying assumptions and articulating competing accounts, researchers can reason more honestly about what their data are able to support.

Sometimes, the bias burden will be too high to sustain a causal claim. But often, careful design, theory‐informed modeling, and sensitivity analysis can meaningfully constrain avoidable and analyst‐induced sources of bias. This reflects Levins (1966) model‐building philosophy for population biology mentioned above, in which “truth is the intersection of independent lies.” With IBE, credible explanation emerges not from any single study's purity, but from the convergence and triangulation of theory, data, and context across multiple imperfect sources.

The critical biocultural synthesis in human biology and anthropology emphasizes local and situated approaches to explaining variation: tracing how bodies are shaped by ecologies, political economies, and structures of power over time (Goodman and Leatherman 1998; Hicks and Leonard 2014; Leatherman and Hoke 2017). In this view, biology emerges not merely alongside social and cultural difference, but through it (Goodman and Leatherman 1998; Jablonka and Lamb 2005). Concepts such as *reactive genomes* (Gilbert 2003; Keller 2014; M. Lock 2017), *emergent embodied phenotypes* (Krieger 2013, 2021), and *biosocial inheritance* (Hoke and McDade 2014) offer analytic scaffolding for tracing how material, social, and historical conditions become embedded in physiological systems.

The spirit of realized causal inference and inference to the best explanation (IBE) aligns with these commitments to theorize the partial and situated nature of both bodies and knowledge. Medical anthropologist Margaret Lock's concept of *local biologies* emphasizes that biology emerges through embodied experience in historically and culturally specific contexts (M. M. Lock 1993). In later work, this evolved into *situated biologies*, which foreground how both human biology and biological knowledge are jointly shaped through situated practices of research, including the institutional, technological, and epistemic conditions under which knowledge is produced (Niewöhner and Lock 2018). These commitments are central to the critical biocultural synthesis in anthropology and human biology.

Taken together, these threads support a vision of anthropological causal inference in human biology not as the discovery of universal laws, but as the generation of contingent, situated explanations of how specific configurations of biology, history, and environment produce particular outcomes. This approach relies on abductive reasoning, which begins with an empirical puzzle and asks what explanation best fits, given what is known, observed, and possible in context. It is a logic of situated causal inference: one that prioritizes plausibility, coherence, and explanatory adequacy over experimental control. The remainder of this paper introduces a structured roadmap for strengthening such inferences in human biology. Drawing on tools from modern causal inference, we outline a principled framework for study design and interpretation that is both empirically rigorous and theoretically grounded.

## 2 A Roadmap for Anthropological Causal Inference in Human Biology

**FIGURE 1:**
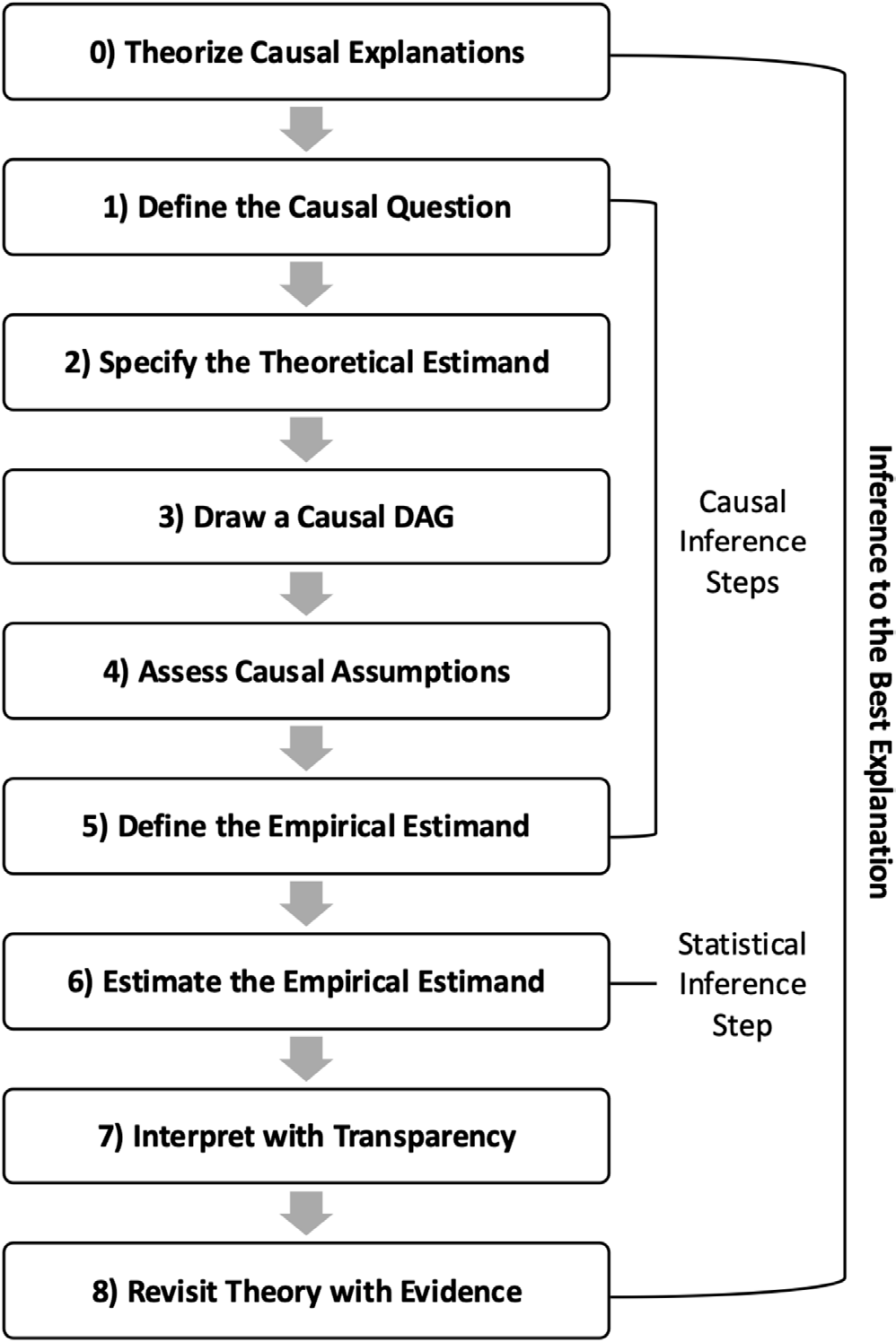
Roadmap for causal inference with observational data.

This tutorial provides a structured roadmap for conducting causal inference using observational data (Figure 1). This process involves defining the causal research question, using causal directed acyclic graphs (DAGs) to clarify the relationships between variables, and evaluating causal inference assumptions before moving on to statistical estimation. To illustrate the fundamental concepts, we focus on binary exposures and total causal effects, which avoid the complexities introduced by continuous exposures^1^ and mediation analysis.

To illustrate each step, we use a running example: estimating the realized effect of being born with low birth weight versus not on inflammation at age 21, using data from the “Local Longitudinal Human Biology Study” (LLHBS), a hypothetical, community‐based study that has followed a representative birth cohort from 1980 to the present.

### 2.1 Step 0: Theorize and Generate Possible Causal Explanations

Causal inference begins with theory, not statistics. Theory and prior evidence helps guide, which explanations are worth testing, what mechanisms are plausible, and how to interpret observed patterns. It anchors hypothesis generation, identifies potential biases, and ensures that inference stays connected to the explanatory goals of human biology. We follow Schwartz and Prins (2025) in anchoring this theory‐first orientation as Step 0 in our causal inference roadmap for human biologists.

*Running Example*: We draw on the Developmental Origins of Health and Disease (DOHaD) framework to hypothesize that intrauterine nutritional constraint, reflected in low birth weight, shapes long‐term immune function. This motivates a causal question about whether individuals born with low birth weight exhibit higher levels of inflammation, as measured by C‐reactive protein, at age 21. Theory also helps anticipate potential confounders, such as socioeconomic conditions or early‐life infections, and informs how we interpret any observed associations.

### 2.2 Step 1: Specify the Causal Question

Once grounded in a substantive theory—and after determining that causal inference, not description or prediction, is the aim—the next step is to clearly articulate the causal research question. What is the exposure? What is the outcome? And where does the causal effect of interest fall along the continuum between idealized, well‐defined interventions and contextually embedded, realized exposures?

*Running Example*: We specify the following research question: What was the realized causal effect of being born with low birth weight (< 2500 g) versus “normal” birth weight (≥ 2500 g) on inflammation (measured via C‐reactive protein levels) at age 21 in the LLHBS?

### 2.3 Step 2: Specify the Theoretical Estimand

To clearly specify the research question, the researcher should next define the *theoretical estimand*: the specific causal quantity of interest defined in terms of counterfactuals and populations, independently of the statistical model or estimator used to approximate it. Drawing on Lundberg et al. (2021), a theoretical estimand has two components: (1) the *unit‐specific quantity*, and (2) the *target population*.

The *unit‐specific quantity* defines what is being measured for each unit (e.g., individual or household) (Lundberg et al. 2021). This can be a *descriptive* quantity, such as the average height in a population, or a *causal effect*, often expressed as a counterfactual contrast of how the outcome would differ for the same unit under two different conditions (e.g., the difference in inflammation if a person had been born with low birth weight versus normal birth weight).

The second component, the *target population*, defines who the estimand refers to (i.e., among whom the unit‐specific quantity is being aggregated) (Lundberg et al. 2021). In human biology and anthropology, this is typically a contextually bounded group: our data often reflect a specific cohort, community, or field site rather than a generic or idealized demographic group (e.g., “all human infants” or “all hunter‐gatherers”). This anchors the estimand in the causal question posed in Step 1 and clarifies the scope of inference. See Box 1 for further discussion of target populations and causal generalization.

BOX 1 **Target populations and causal generalizations.**

Further assumptions are required if the researcher aims to generalize from a single study to similar or different populations. As Degtiar and Rose (2023) note, assessing the validity of such generalizations involves clarifying the target population we wish to generalize to and identifying potential sources of external validity bias, including treatment effect heterogeneity and differences in population structure. Transporting a causal effect estimate to a different target population requires strong assumptions: that the distribution of effect modifiers differs in known and correctable ways, and that the exposure itself retains consistent construct validity across contexts.

Unlike interventional causal inference, where well‐defined treatments make such assumptions more plausible, realized causal inference engages with historically embedded, socially patterned exposures whose meaning is deeply context‐dependent. Critics of what they term the “historicist's refuge” argue that confining inference to a single study context limits the ability to build deductive generalized causal knowledge (Esterling et al. 2025). Yet in realized causal inference, the study population *is* often the target population by design. Nevertheless, careful consideration of exposure construct validity and how the distribution of relevant effect modifiers varies across contexts is critical for synthesizing across individual studies.

From an IBE perspective in human biology, this resonates with an abductive mode of generalization (through the accumulation and comparison of evidence across studies) rather than solely extrapolating a causal effect estimate from a single study to a more general population.

Most commonly, researchers aim to estimate an average treatment effect (ATE), which summarizes the average causal effect of exposure versus non‐exposure across the entire study population. This is often the most intuitive estimand, especially when the goal is to understand overall population‐level effects. In some cases, an average treatment effect on the treated (ATT) may be of interest, but for clarity and accessibility, we focus primarily on ATE in this guide. Readers interested in alternative estimands can consult additional resources for further exploration (Desai and Franklin 2019; Heiss 2024; Wang et al. 2017).

*Running Example*: Our theoretical estimand could be defined as a realized average causal effect, analogous to an ATE, in the LLHBS of being born with versus without low birth weight on inflammation at age 21. Formally, this can be written as:

$$\psi =Ε\left[Y_{i}^{1}-Y_{i}^{0}\right]$$

where ψ is the realized average causal effect in this population. E[.] is the expectation (or average) taken over all individuals in our target population. For a realized causal effect, the target population is our specific sample under study within its historical context. $Y_{i}^{1}$ is the potential outcome (CRP level at age 21) for individual i if they had been born with low birth weight (< 2500 g). $Y_{i}^{0}$ is the potential outcome for individual i if they had been born without low birth weight (< 2500 g).

### 2.4 Step 3: Draw a DAG to Ground Reasoning About the Relationships Between Variables of Interest

Causal DAGs encode our assumptions by visually representing the direction of the causal pathways theorized to exist between variables in a system under study (Pearl 2021). Subject matter expertise plays a critical role here, as the assumptions encoded in a DAG are grounded not in statistical correlations within the analytic data, but rather in a priori understanding of the causal system informed by a synthesis of extant literature, theory, and insights from fieldwork. This is critical because different causal structures can produce identical statistical associations; relying on correlations alone to guide adjustment decisions can therefore yield seriously biased results. McGowan et al. (2023), for example, demonstrate how the same regression coefficient can arise under completely distinct data‐generating mechanisms (including confounding, mediation, collider stratification bias—each defined below) and that correct causal inference depends on understanding these underlying structures, not statistical summaries alone.

A DAG consists of two fundamental building blocks: nodes and edges. *Nodes* represent the variables in the system being modeled, while *edges* (the arrows connecting nodes) represent the directed flow of causation from one variable to another.

DAGs are directed and acyclic: they contain no double‐headed arrows, and no variable can cause itself. In this way, DAGs formalize the assumption that causes flow forward in time, and temporal ordering is respected. When a time‐varying process is of interest (such as repeated measures of the exposure, confounders, or outcome), it can be represented by multiple nodes, with unidirectional arrows representing each time period.

**FIGURE 2:**
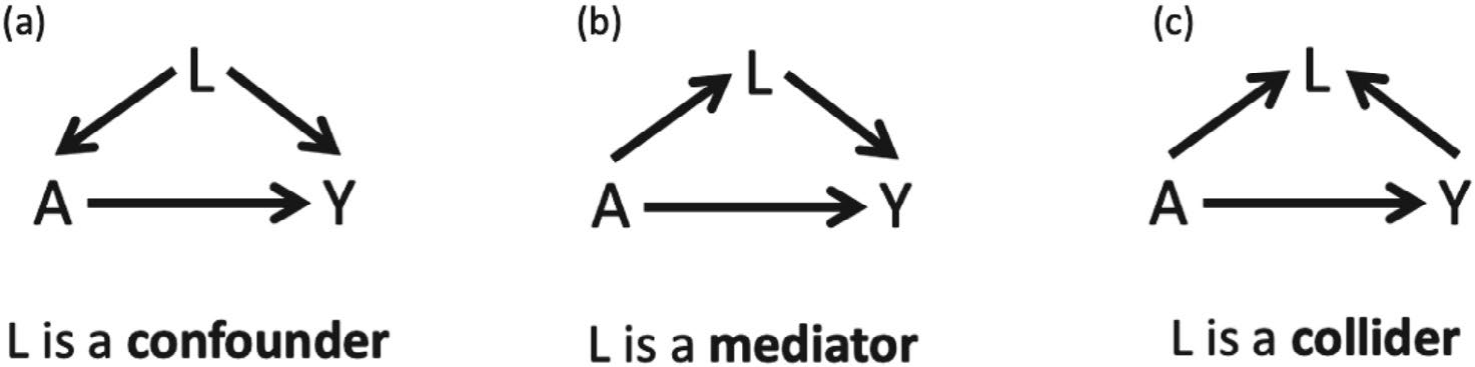
The three fundamental structures on a DAG representing possible relationships between an exposure, A, an outcome, Y, and a third variable in a causal system, L.

There are three fundamental structures on a DAG to recognize (Figure 2):

- *Confounder* (Figure 2a) variables are *pre‐exposure* variables that are known or plausible *causes* of both the exposure *and* outcome. Confounding is a causal concept, not a statistical concept (Hernán et al. 2002). Therefore, bivariate associations in the analytic dataset between a covariate and the exposure or outcome should not guide DAG construction because associational evidence does not indicate causal direction or ordering. Instead, confounding variables should be identified using a priori expert knowledge, drawing from a critical synthesis of the literature, theory, and insights from fieldwork.
- *Mediator* (Figure 2b) variables are post‐exposure variables that act as mechanisms, which explain a portion of the exposure's causal effect on the outcome. Although not depicted in the figure, mediators are often also colliders due to measured or unmeasured confounders affecting the mediator–outcome relationship (discussed further below).
- *Collider* (Figure 2c) variables are post‐exposure variables that are caused by the exposure and caused by the outcome. Colliders are labeled as such because they are present when a directed arrow coming from the exposure and a directed arrow coming from the outcome “collide.” Although the figure depicts a collider connected by direct paths from exposure and outcome, collider paths can also originate from arrows extending from nodes that are upstream or downstream of either the exposure or the outcome. Selection bias is a common real‐world example of collider bias, where inclusion in the analytic sample depends on variables influenced by both the exposure and the outcome (see Box 2).

BOX 2 **Selection bias, collider bias, and DAGs.**

A powerful insight from DAG‐based causal reasoning is that selection bias is a form of collider bias: it emerges when we include or exclude participants from a study based on a variable that is influenced by both the exposure and the outcome, or by their shared causes (Hernán et al. 2004; Lu et al. 2024).

Attrition, or loss to follow‐up, in observational research provides a canonical example (Howe et al. 2016). Imagine a study estimating the effect of elevated depression symptoms on cognitive function in midlife. Suppose both depression and cognitive decline influence whether participants attend the follow‐up visit. If we restrict analysis to only those retained in the study, we implicitly condition on follow‐up status, which is a downstream effect of both exposure and outcome. This creates a noncausal path between depression and cognition, biasing the estimate even if all baseline confounders are adjusted for. This can be illustrated in this simplified DAG:

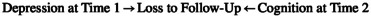

One solution is to model the selection process directly using inverse probability of censoring weights (IPCW), which reweight individuals based on their estimated probability of being retained in the sample, given covariates that predict attrition (Robins and Finkelstein 2000; Weuve et al. 2012). When correctly specified, IPCW can help recover unbiased effect estimates by creating a pseudo‐population in which censoring is independent of both exposure and outcome. However, IPCW requires strong assumptions: no unmeasured predictors of loss to follow‐up, correct model specification, and sufficient overlap in covariate distributions. DAGs can help researchers assess whether these assumptions are plausible by explicitly representing the causal structure of selection. Other missing data approaches, such as multiple imputation by chained equations (MICE), can also be used to address selection bias and other forms of missing data (Shaw et al. 2023).

Rather than treating attrition as an unavoidable limitation, causal diagrams invite us to see it as part of the data‐generating process and thus a core concern of study design, causal inference, and statistical analysis.

Of note, DAGs represent only the presumed direction of causation between variables and cannot formally directly depict hypothesized effect modification or moderation across covariates. However, effect heterogeneity should be carefully considered during the statistical estimation phase, as is briefly discussed in Step 6 below.

#### 2.4.1 Drawing a Causal DAG Begins With Adding the Exposure and Outcome Nodes

**FIGURE 3:**
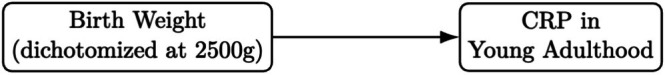
Incomplete causal DAG with exposure (birth weight) and outcome (inflammation) nodes.

Drawing a DAG begins with adding nodes for the *exposure* and *outcome* variables (Figure 3). As mentioned above, DAGs should only correspond to one exposure and outcome pair. Even within similar research questions using the same dataset, a variable may be a confounder for one research question but a mediator for another. Therefore, if several exposures or outcomes are of interest, separate DAGs should be drawn for each exposure‐outcome pair to ensure understanding of the specific causal relationships at play.

#### 2.4.2 Next, Use Subject Matter Expertise to Identify and Add Confounding Variables (i.e., Pre‐Exposure Factors That Cause Both the Exposure and Outcome) to the DAG

This step relies on expert knowledge rather than statistical algorithms, model selection processes, or correlations observed in the analytic data. Confounders open what is called a “backdoor path” on a DAG, where a chain of variables starts at the exposure, passes through other variables, and ends at the outcome. These backdoor paths are directly related to the assumption of exchangeability (discussed in Step 4). If left unblocked, confounders create systematic differences between the exposed and unexposed groups, violating exchangeability and leading to biased causal estimates.

To achieve exchangeability, all open backdoor paths must be blocked by adjusting for confounders, while taking care to avoid conditioning on collider variables. As will be discussed further below, this is because the presence of a collider node *closes* a backdoor path. In contrast to the case for confounding variables, statistically adjusting for a collider variable unnecessarily *opens* a spurious backdoor path that would have otherwise been closed, introducing potential bias. Researchers often routinely adjust for variables that are merely conceptually related or statistically correlated with the exposure and outcome without considering their causal relationships or ordering. By visually representing assumptions about the relationships between variables, DAGs help researchers reason systematically about their assumptions regarding which variables to adjust for, and clearly present these assumptions to the research community.

**FIGURE 4:**
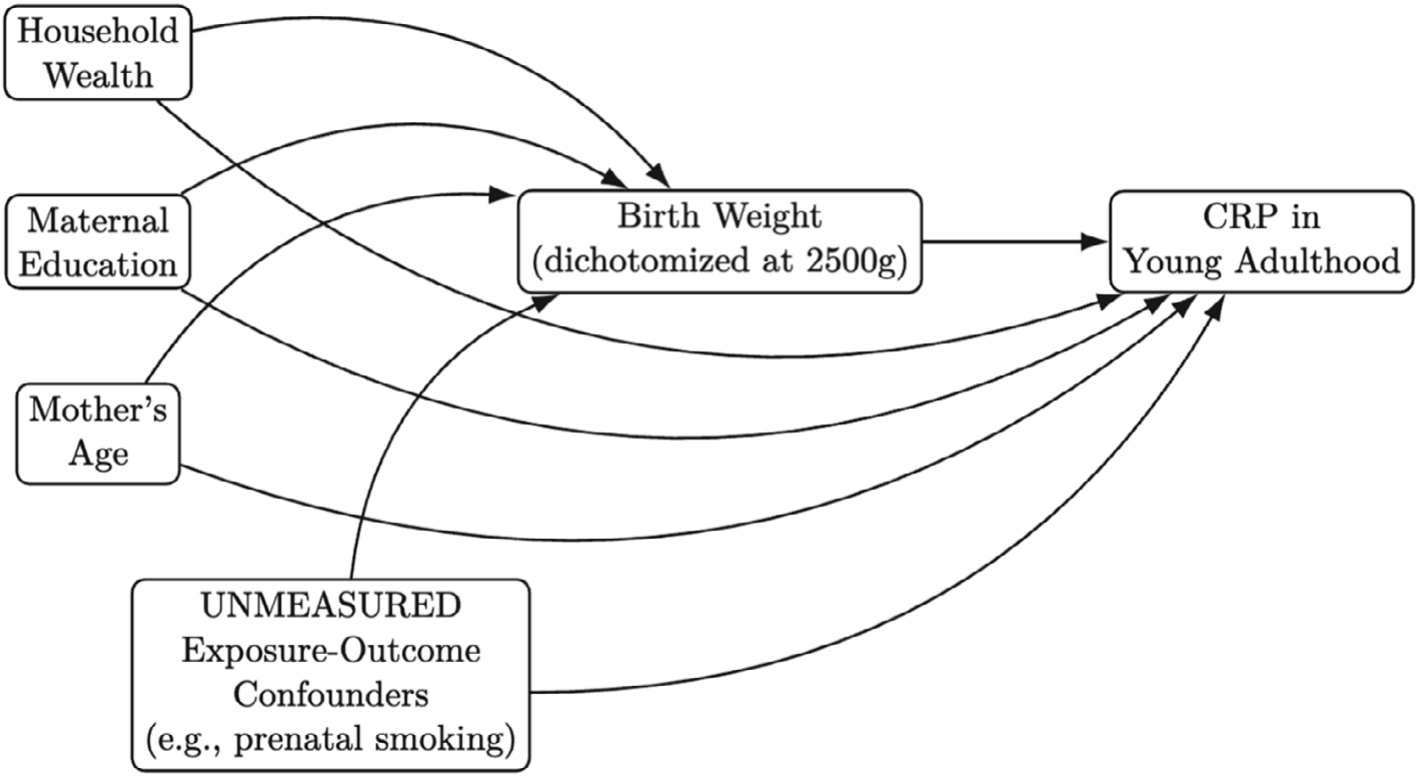
Causal DAG with exposure (birth weight), outcome (inflammation), and confounder nodes.

*Running Example*: We update our example DAG below (Figure 4) to add maternal age, maternal education, and household wealth as plausible pre‐exposure confounders that may influence both birth weight and inflammation in adulthood. Maternal age can impact pregnancy outcomes and future child health through effects on fetal development (Barclay and Myrskylä 2016; Duncan et al. 2018; Fall et al. 2015). Maternal education and household wealth reflect socioeconomic resources, which are associated with both birth weight and inflammation risk (Blumenshine et al. 2010; Milaniak and Jaffee 2019). While many more known plausible confounders exist and would be needed in a real study, we present only these for simplicity. To ensure transparency and clearly communicate the limitations of our causal assumptions, we include a node representing unmeasured confounding (i.e., common causes of the exposure and outcome that are unknown or not fully specified). This signals that the DAG does not depict a fully identifying structure, but instead more truthfully reflects the partial and provisional nature of our knowledge about the data‐generating process.

#### 2.4.3 Though They Need Not Be Added for a DAG to Be Complete, Be Vigilant in Recognizing and Not Adjusting for Post‐Exposure Variables (i.e., Mediators and/or Colliders)

In human biology and other social sciences, researchers sometimes inadvertently introduce avoidable bias by statistically adjusting for variables that are not true confounders, a practice economists often refer to as adjusting for “bad controls” (Angrist and Pischke 2009; Cinelli et al. 2020). It is important to carefully consider post‐exposure variables like mediators and colliders, as conditioning on them during analysis can introduce bias. Even if two variables are truly independent, once you condition on a common effect (the collider), it can introduce bias in at least one stratum of the collider (Banack, Mayeda, Naimi, et al. 2024). This phenomenon is known as *collider stratification bias*.

While confounders lie on open backdoor paths on a DAG and need to be adjusted for to remove bias, colliders by nature *block* backdoor paths and should not be adjusted for. Adjusting for a collider opens a previously closed backdoor path, leading to an analyst‐induced source of spurious association between the exposure and the outcome. The magnitude and direction of collider stratification can be difficult to predict or intuit, but generally the magnitude of bias is greater when the collider is directly caused by the exposure and outcome rather than through variables that are downstream descendants of the exposure and/or outcome (Banack, Mayeda, Fox, et al. 2024).

Selection bias is a particularly important form of collider bias. It arises when inclusion in the analytic sample depends on a variable influenced by both the exposure and the outcome (or by their shared causes), thereby inducing a noncausal association even when confounders are properly adjusted for. Because selective attrition is common in longitudinal studies, it is critical to represent this explicitly in DAGs. Box 2 illustrates this principle through a concrete example of attrition in a study of depression and cognitive decline and outlines analytic strategies for addressing it.

Similarly, adjusting for mediators not only removes part of the total causal effect but may also introduce unnecessary collider bias through unmeasured common causes of the mediator and outcome. This form of bias is often labeled as post‐treatment bias, post‐exposure bias, or overadjustment bias (Cole and Hernán 2002; Kaufman et al. 2004; Montgomery et al. 2018; Schisterman et al. 2009; van Zwieten et al. 2022). While mediation questions are often of interest, researchers should first be able to rigorously estimate the *total* causal effect before moving on to estimate direct and indirect effects, which require more stringent assumptions about the direction and ordering of variables in a system under study (Stuart et al. 2022).

**FIGURE 5:**
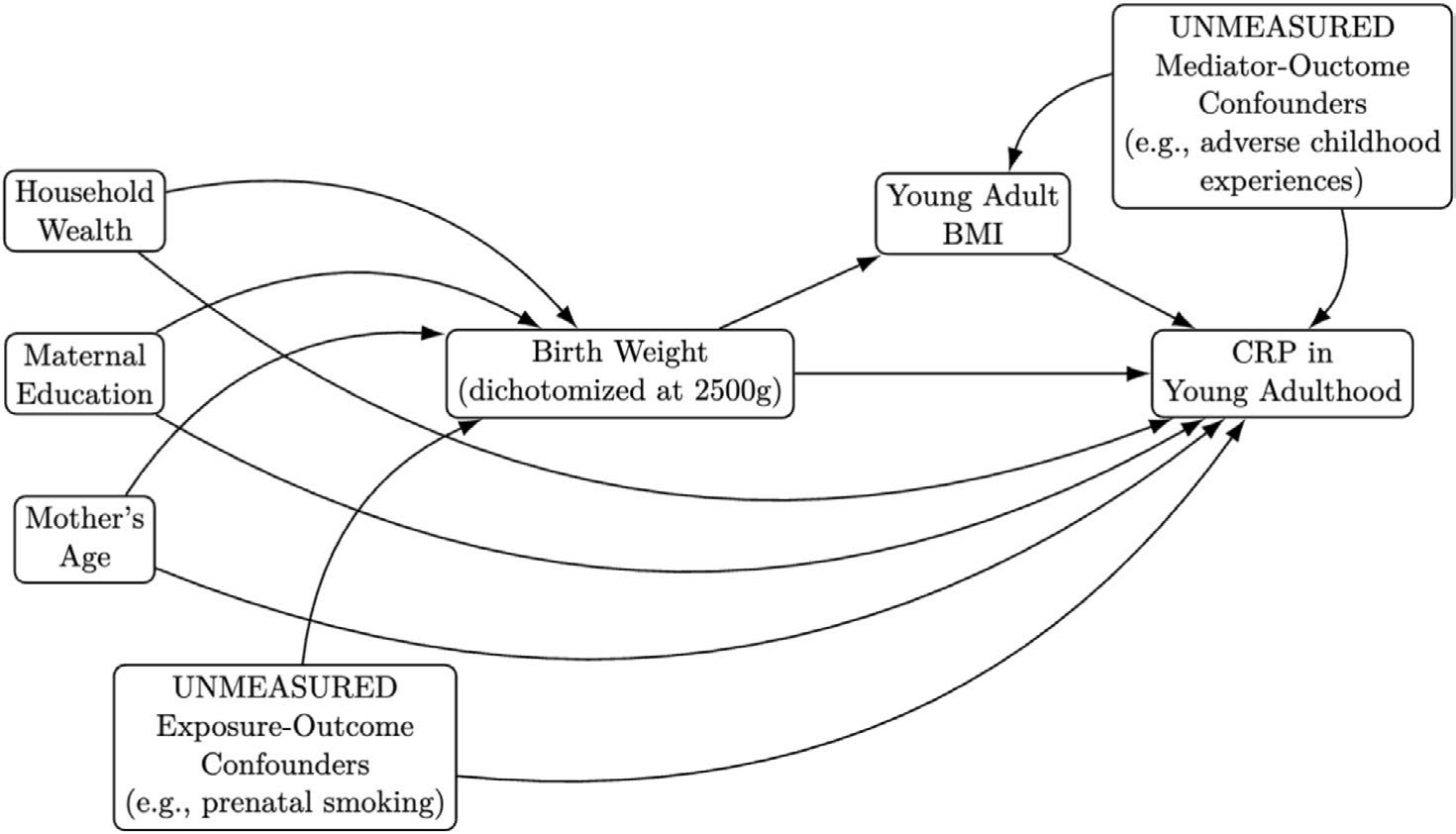
Extended causal DAG showing mediation path including unmeasured mediator–outcome confounders.

*Running Example*: Despite evidence that BMI likely impacts CRP levels, BMI in young adulthood should not be adjusted for in the analysis of the effect of low birth weight on inflammation at age 21 because it functions as both a mediator and a collider in this setting (Figure 5). BMI in adulthood is influenced by both low birth weight and other unmeasured factors that affect inflammation, such as adverse childhood experiences. Thus, conditioning on adult BMI would thus remove a portion of the total effect while simultaneously introducing collider stratification bias, potentially biasing the association between birth weight and inflammation in adulthood.

### 2.5 Step 4: Evaluate Causal Assumptions to Identify Whether the Theoretical Estimand Can Be Linked to an Empirical Estimand That is a Parameter of the Observed Data

Assumptions are required for realized causal inferences with observational data. For both realized causal effects and interventional causal effects, these include *conditional exchangeability*, *positivity*, *non‐interference*, and *construct validity*. Interventional causal effects additionally require evaluating the *consistency* assumption, a particular type of construct validity focused on a clear and precise mapping between the exposure category definitions and the (potentially hypothetical) intervention on the exposure.

#### 2.5.1 Conditional Exchangeability

When exchangeability holds, we expect similar outcomes on average in the absence of exposure if we were to swap members of the exposed and unexposed groups (Greenland and Robins 1986). Exchangeability is achieved in randomized trials because randomization renders treatment assignment independent of all participant characteristics, ensuring comparability (on average across multiple hypothetical trial samples) at baseline across both measured and unmeasured characteristics.

However, in observational studies, we must instead assess the assumption of *conditional* exchangeability, which relies on statistical adjustments for measured confounders and the assumption that there are no unmeasured confounders. This assumption is inherently untestable using the data alone. It must be justified using subject‐matter knowledge and theory about which covariates are sufficient to block backdoor paths on a DAG between the exposure and the outcome.

In addition to confounder‐adjustment strategies, which we focus on in this article, there are also design‐based or “natural experiment” approaches to causal inference that leverage exogenous or “as good as random” exposures to facilitate causal inference without the need for confounder adjustment. We briefly introduce quasi‐experimental approaches after the roadmap in our broader reflection on causal inference in human biology.

#### 2.5.2 Positivity

Positivity requires that for every combination of confounders, there is a non‐zero probability of receiving each level of the exposure (Zivich et al. 2022). That is, the exposure must vary across confounder strata to permit meaningful comparisons.

Violations of this assumption fall into two categories: deterministic and stochastic (Zivich et al. 2022). *Deterministic positivity violations* occur when it is biologically or logically impossible for some individuals to receive a given exposure. For example, individuals born without a uterus cannot undergo hysterectomy. In such cases, the causal effect is undefined for those strata, reflecting a fundamental limit of identifiability. *Stochastic positivity violations* arise when certain exposure–confounder combinations are theoretically possible but, by chance, are rare or absent in the data.

Unlike conditional exchangeability, positivity can be partially assessed using observed data. Ensuring sufficient overlap is essential for valid comparisons and interpretation of estimated effects. These issues may sometimes be addressed through modeling strategies (e.g., weighting), or by restricting the sample to areas of covariate overlap. However, such strategies redefine the estimand and limit inference to a subpopulation with sufficient overlap.

#### 2.5.3 No Interference

This assumption requires that one individual's exposure level does not affect another individual's outcome. In human biology, violations of non‐interference might arise in contexts where one person's exposure could influence another person's outcome, as might happen with contagious disease exposures. In such situations, specialized causal inference and statistical tools that account for network or spillover effects are necessary, but those approaches are beyond the scope of this article. For an overview of causal inference in the presence of interference see Hudgens and Halloran (2008) or Tchetgen and VanderWeele (2012) and for a recent applied example of causal social network analysis from social epidemiology see Makofane et al. (2023).

#### 2.5.4 Causal Consistency

Causal consistency is required only when estimating interventional causal effects, that is, when asking what would happen under a well‐defined manipulation of the exposure. The assumption holds that the observed outcome under an exposure level corresponds to the potential outcome that would be observed if that exposure were assigned under a well‐defined intervention. Importantly, consistency does not require that all individuals experience the exact same version of the exposure, but rather that any variation in the exposure's form does not lead to meaningful variation in its effect on the outcome (Cole and Frangakis 2009; Pearl 2010; T. J. VanderWeele 2009; VanderWeele and Hernán 2013). Causal consistency and non‐interference together comprise the Stable Unit Treatment Value Assumption (SUTVA) (Rubin 1980).

While easier to ensure in randomized trials—or when observational studies are able to closely emulate them—consistency is often difficult to satisfy in human biology. Socially embedded exposures like food insecurity, stress, or mental health are frequently operationalized through composite measures that collapse heterogeneous experiences into a single index. If these different exposure components affect outcomes differently, the consistency assumption is violated. In such cases, researchers may consider disaggregating composite scales into subscales or individual items to better approximate narrower, yet perhaps more tractable hypothetical interventions (for a discussion of evaluating the consistency assumption with social exposures, see Rehkopf et al. 2016).

#### 2.5.5 Consistency and Construct Validity in Realized Causal Inference

In realized causal inference, the formal assumption of causal consistency plays a different role than in interventionist frameworks. Because the goal is to estimate what an exposure did within a particular historical, social, and biological context, variation in how the exposure was instantiated across individuals is treated as part of the effect being summarized (Schwartz et al. 2016; Schwartz and Prins 2025). Thus, construct validity becomes a central concern: researchers must ensure that the exposure is conceptually coherent, theoretically grounded, and meaningful in the study context. However, this does not mean that concerns about consistency can be ignored altogether. In realized causal inference, researchers can consider consistency not as a strict assumption, but as a lens for understanding heterogeneity in how an exposure operates. For example, a composite construct like a food insecurity experiences index may produce similar total scores across individuals while obscuring different underlying experiences, such as limited availability, poor access, or disrupted utilization. Rather than relying solely on a high vs. low cutoff, researchers might also explore how specific scale subdomains relate to outcomes, clarifying what the realized causal effect is actually summarizing.

In addition to construct validity, measurement error in the exposure, outcome, and other variables in the causal diagram is also important to consider. DAGs can be used to reason explicitly about random, systematic, and differential measurement error, including cases where the error depends on other nodes in the graph (VanderWeele and Hernán 2012). Bulbulia (2024c) offers a comprehensive overview of these issues, using DAGs to show how measurement and selection processes can structurally distort causal inferences, particularly in cross‐population comparative studies. While a full treatment of measurement bias is beyond the scope of this roadmap, researchers should engage with these concerns as part of their broader causal reasoning.

#### 2.5.6 My Assumptions Are (not) Fully Met. What Now?

If the core causal assumptions are met (i.e., exchangeability, positivity, no interference, and consistency or exposure construct validity), then the resulting estimate can be interpreted as a causal effect. But in most real‐world settings, especially with observational data, these assumptions are often not fully satisfied.

Even when causal assumptions are not fully met, researchers may still proceed with estimation, but they should do so transparently (Petersen and van der Laan 2014). It is not enough to label findings as “associational” and move on without clearly conveying how causal assumptions were partially met or unmet (Hernán 2018). Clarifying which assumptions may be violated, how that affects interpretation, and what kinds of bias may result helps ensure that studies with imperfect identification can still contribute to inference to the best explanation. Multiple sources of evidence, each with limitations, may together strengthen a coherent and credible causal account.

*Running Example*: In our illustrative example examining the effect of low birth weight on inflammation at age 21, we would take care to include all measured pre‐exposure confounders on the DAG, and we would consider and plan for sensitivity analyses to assess the impact of unmeasured confounding. We would assess positivity by ensuring sufficient variation in covariates across the binary exposure groups (low birth weight vs. not). We could probe the plausibility of the consistency assumption and assess construct validity by exploring alternative cut‐offs for defining “low birth weight.” To address selection bias from loss to follow‐up, we would examine selective attrition from the study using a DAG and plan to address this analytically. Finally, we assume no interference, which is reasonable given the individual‐level biological nature of the exposure and outcome. Transparent consideration of these assumptions supports cautious interpretation and strengthens inference to the best explanation.

### 2.6 Step 5: Specify the Empirical Estimand

After reasoning through the causal assumptions, the next step is to translate the theoretical estimand into an *empirical estimand*, a quantity defined in terms of observed data that approximates the causal contrast of interest (Lundberg et al. 2021). This operationalizes the theoretical contrast and allows us to reason about causal estimands under real‐world data conditions. Importantly, the empirical estimand can be non‐parametric: it need not assume any particular statistical model or functional form. Those choices enter later, during estimation.

*Running Example:* Recall that our theoretical estimand is:

$$\psi =Ε\left[Y_{i}^{1}-Y_{i}^{0}\right]$$

This represents the realized average causal effect of being born with low versus normal birth weight on inflammation in young adulthood. There are several options for translating this quantity into an empirical estimand that can be estimated with observed data. Here, we show one suitable option:

$$\hat{\psi }=E_{X}\left[Ε\left[Y|A=1,X\right]-Ε\left[Y|A=0,X\right]\right]$$

where $\hat{\psi }$ is the estimated realized average causal effect of low birth weight on inflammation in adulthood in the LLHBS. The outer empirical expectation, E_x_[.], is taken over the observed distribution of confounding variables, resulting in an average causal effect estimate (rather than a conditional effect where confounders are all set to a constant value). Within the outer expectation, E[.] are the empirical expectations taken over those in the data who were exposed (*A* = 1) or not (*A* = 0). *Y* is the outcome (log‐transformed CRP level at age 21). *A* is the binary exposure (1 = low birth weight, 0 = normal birth weight). X is a vector of measured pre‐exposure confounders selected using the DAG from Step 3.

### 2.7 Step 6: Estimate the Empirical Estimand Using an Appropriate Statistical Model

Next, we estimate the empirical estimand from step 5. This is a statistical inference task, not a causal inference one. The causal interpretation depends entirely on the design and assumptions laid out in prior steps, not the method itself (Freedman 1991). Even simple methods like t‐tests can yield valid causal estimates under randomization. But, in observational studies, valid causal inference requires accounting for confounding by covariate adjustment or study design (Hernán and Robins 2020; Imbens and Rubin 2015; Morgan and Winship 2007; Pearl 2021). Either frequentist or Bayesian statistical approaches may be used for estimation; neither is inherently more appropriate for causal inference. In what follows, we focus on common approaches in the frequentist tradition, such as g‐computation and propensity score methods. For more on Bayesian modeling for causal inference with observational data, see McElreath (2020).

This confounding adjustment must be guided by a causal model, not data‐driven variable selection. In our roadmap, this would mean that, ideally, all confounders identified using a directed acyclic graph (DAG) are measured and included in the statistical analysis. Covariate selection algorithms (e.g., stepwise regression) or model fit metrics (e.g., R‐squared or AIC) should not be used to determine which variables to adjust for, as they are agnostic to the structural roles of variables on a DAG (e.g., confounder, mediator, collider). That is, including a mediator or collider may improve model fit, but could be disastrous from a causal perspective (for more information on this, see Arif and MacNeil 2022 and McElreath 2020).

#### 2.7.1 Augment Familiar Outcome Regression to Recover Marginal Effects

In human biology, the most commonly used estimator is outcome regression, where the outcome is modeled as a function of the exposure and covariates. This includes generalized linear models (e.g., linear and logistic regression) and their extensions to multilevel data structures. These models estimate *conditional associations*, that is, effects within levels of the covariates that are “held constant.” To recover valid causal effects, all confounders identified in the DAG should be included, and effect modification should be accounted for by including exposure–covariate interaction terms when theory or prior evidence suggests heterogeneity.

However, causal inference and anthropological reasoning rarely operate in a world where confounders are held constant. We are typically interested in *marginal* (*or average*) *associations and effects*, that is, population‐level contrasts that reflect how outcomes differ, on average, across levels of exposure (Arel‐Bundock et al. 2024). These align more closely with the realized causal effects described earlier in the roadmap. To estimate marginal effects using outcome regression, researchers can apply *g‐computation*, which uses a fitted regression model to predict potential outcomes under each exposure level and averages them across the observed covariate distribution of the relevant target population, as defined in Steps 2 and 5. See Snowden et al. (2011) for an accessible overview of g‐computation and Arel‐Bundock (n.d.) for an implementation tutorial in R via the “marginaleffects” package (Arel‐Bundock et al. 2024).

An alternative approach is to use *propensity score methods*, which model the probability of exposure given covariates (Rosenbaum and Rubin 1983, 2022). This allows for confounder adjustment without explicitly modeling outcome heterogeneity. Methods such as *inverse probability of treatment weighting* (*IPTW*) reweight observations using the propensity score so that the treated and untreated groups are comparable on observed confounders (Cole and Hernán 2008). These weights can then be used in a bivariate regression of the exposure on the outcome to estimate a marginal effect, taking care to use robust or bootstrapped standard errors to account for the regression weights. For a comprehensive introduction to causal inference with propensity score methods and inverse probability weighting, see Hernán and Robins (2020).

#### 2.7.2 Statistical Inference Beyond *p* < 0.05

We echo calls within human biology and across the social sciences to move away from using *p* values as rigid significance thresholds (Amrhein et al. 2019; Amrhein and Greenland 2022; Gelman and Stern 2006; Valeggia and Fernández‐Duque 2022). Instead, we encourage researchers to interpret the magnitude of the effect and the width of the uncertainty interval in relation to biological relevance and the plausibility of both causal and statistical modeling assumptions.

#### 2.7.3 Conduct Sensitivity Analyses That Probe Statistical and Causal Assumptions

Where feasible, researchers should evaluate how violations of key assumptions—both causal and statistical—could affect their conclusions. This includes assessing the potential impact of unmeasured confounding, model misspecification, violations of positivity, missing data, selection bias, or measurement bias.

Sensitivity analysis for unmeasured confounding is a central component of quantitative bias analysis (QBA), a set of methods used to assess how violations of causal assumptions, such as conditional exchangeability, might affect causal effect estimates (Kawabata et al. 2023). Some of these approaches, like the *E*‐value, do not correct for unmeasured confounding but instead improve transparency by clarifying the strength or structure of bias required to change a study's conclusions. The *E*‐value quantifies the minimum strength of association that an unmeasured confounder would need to have with both the exposure and outcome (conditional on measured covariates) to explain away the observed effect (VanderWeele and Ding 2017; VanderWeele and Mathur 2020). While widely adopted in epidemiology, the *E*‐value is limited to the risk ratio scale and can be difficult to interpret for continuous outcomes or common exposures.

Other tools go further by allowing researchers to specify assumptions about the strength of unmeasured confounding and adjust effect estimates accordingly. For example, the R package *sensemakr*, developed by Cinelli and Hazlett (2020), enables users to estimate adjusted coefficients and confidence intervals for linear models under hypothesized confounder–treatment and confounder–outcome relationships. It offers partial *R* ^2^ metrics, robustness values, contour plots, and benchmarking tools to make explicit how strong an unmeasured confounder would need to be in order to materially change interpretation. Tools like the *E*‐value and those implemented in *sensemakr* reflect a broader class of diagnostic strategies for evaluating the credibility of causal inferences under imperfect identifiability. We recommend their use in conjunction with careful study design and subject‐matter expertise, not as replacements for identification, but as complements to transparent, assumption‐aware analysis.

*Running Example*: In our hypothetical study, we could use a weighted linear regression to estimate the realized effect of low birth weight on C‐reactive protein levels in the LLHBS, using inverse probability of treatment (IPTW) and censoring (IPCW) weights to adjust for the pre‐exposure covariates identified on the DAG to reduce bias from confounding and selection bias. To evaluate the robustness of our findings to potential unmeasured confounding, we could then calculate the E‐value and apply sensitivity analysis tools such as those in the *sensemakr* R package.

### 2.8 Step 7: Transparently Interpret the Effect Estimate in Light of Causal Assumptions

Interpretation should consider how well the causal assumptions were met. When assumptions hold strongly, a causal interpretation may be warranted. If the assumptions were only weakly met, results should be interpreted cautiously as associations rather than causal estimates.

Still, estimates derived under imperfect conditions are not without value. By clearly defining the causal estimand and mapping where assumptions hold or break down, researchers can provide transparent, structured interpretations that retain explanatory utility. This enables the study to inform inference to the best explanation, in which evidence from multiple imperfect sources, each with clearly stated limitations, contributes to adjudicating among competing causal accounts.

It is also important to distinguish between the exposure of interest and other coefficients when estimating causal effects using a multiple regression model. While commonly reported and interpreted, confounder coefficients should not be interpreted as meaningful. These coefficients are conditional on the exposure of interest, which is by definition a downstream effect of the confounders, thus making the covariate coefficient conditional on a mediator and potentially introducing collider bias. As a result, these estimates are not inferentially meaningful and can be easily misinterpreted. The widespread naïve interpretation of covariate coefficients has been termed the “Table 2 Fallacy” (Westreich et al. 2021; Westreich and Greenland 2013). If interest in a covariate–outcome relationship arises, it should be treated as a distinct causal question and addressed by returning to Step 0 of the roadmap.

Within an IBE approach, a study can still contribute meaningfully even if it does not meet all causal identification criteria (Spirling and Stewart 2024). What matters is that researchers clearly specify what the estimate represents, the assumptions it relies on, and how those assumptions affect interpretation. Such transparency enables human biologists to build cumulative, theory‐driven explanations of biosocial processes across diverse and imperfect evidence.

### 2.9 Step 8: Revisit the Theory in Light of the New Evidence

This final step explicitly grounds the roadmap within inference to the best explanation. After defining a causal question, designing an analysis, and estimating an effect, researchers should return to the substantive theory that initially motivated the study (Step 0) and evaluate how well the findings support it. The goal is not simply to report a statistically significant association, but to assess whether the total body of evidence increases or decreases our confidence in the proposed explanation.

This involves asking: Do the findings align with theoretical expectations from anthropology, biology, or social science? Are alternative explanations such as confounding, measurement bias, or reverse causation plausibly ruled out? Do the results fit into a coherent causal narrative about how the exposure contributed to the observed outcome, in this setting, at this time?

Beyond theoretical alignment, researchers should consider the biological or practical meaningfulness of the result. Is the effect large enough to matter physiologically or clinically? Does it plausibly operate through known mechanisms? For human biologists, this may include reflecting on how findings intersect with developmental processes, ecological pressures, or evolutionary tradeoffs.

Finally, findings can be contextualized within other forms of evidence. For multi‐method researchers, this might involve drawing on ethnographic data, historical context, or parallel analyses in related populations. Integrating these sources strengthens the explanatory power of the causal claim and situates it within a broader empirical and theoretical landscape.

## 3 Opportunities for Enhancing Rigor and Analytical Ethics in Human Biology

In this toolkit paper, we have described how human biologists can apply tools from contemporary causal inference to our work, even if the nature of our research does not always neatly meet all causal inference assumptions. Beyond the roadmap provided above, we now broaden our scope to discuss opportunities for enhancing rigor and analytical ethics in human biology.

### 3.1 Alternative Routes to Examining Mechanisms Without Formal Causal Mediation Analysis

Mechanisms are central to many questions in human biology. Researchers often want to understand *how* an exposure produces an outcome, not just whether it is associated with an outcome net of confounders. Formal causal mediation analysis is one approach for elucidating such pathways, but it comes with stringent assumptions and requires advanced statistical methods not covered in this article. These assumptions include no unmeasured confounding of either the exposure–mediator or mediator–outcome relationship, correct specification of any exposure–mediator interactions, and careful handling of mediator‐outcome confounders that are downstream effects of the exposure (Cole and Hernán 2002; Stuart et al. 2022; T. VanderWeele 2015). In light of these numerous assumptions for causal mediation, widely used approaches like the Baron and Kenny mediation model are now recognized as inappropriate under many real‐world data conditions facing human biology and other social sciences (Stuart et al. 2022).

We encourage human biologists to first develop comfort with estimating total causal effects using the roadmap presented here. Gaining experience with specifying an estimand, using DAG‐based reasoning, and selecting an estimator that is appropriate for the estimand lay the groundwork for more complex mediation analyses.

As a more tractable alternative, researchers can examine intermediate variables as outcomes in their own right. For example, if adult BMI is thought to mediate the relationship between low birth weight and inflammation, one could use the roadmap to estimate a total effect of low birth weight on adult BMI. This sheds light on plausible pathways without the burden of full mediation assumptions. While such analyses do not decompose effects into “direct” and “indirect” components, they still contribute meaningfully to our understanding of embodied mechanisms.

For those interested in formal causal mediation analysis, we recommend Nguyen et al. (2021), Schuler et al. (2024), and Stuart et al. (2022) as accessible introductions.

### 3.2 Beyond Confounder Adjustment: Quasi‐Experimental Approaches

This paper has focused on confounder‐control methods appropriate for estimating realized causal effects in the kinds of data that define much of human biology: long‐term, field‐based, community cohort studies rich in theoretical framing and local contextual detail. These designs offer unique advantages for understanding causality in situated human populations. However, an important set of tools in the causal inference literature grouped under the umbrella of quasi‐experimental designs provides alternative and complementary strategies for identifying and estimating causal effects with observational data. These include instrumental variable (IV) analyses, regression discontinuity (RD) designs, mendelian randomization (MR), and difference‐in‐differences (DiD) frameworks, all of which rely on the presence of exogenous variation in an exposure, rather than comprehensive confounder measurement, to estimate causal effects (Angrist and Pischke 2009; Imbens 2024; Matthay et al. 2019).

Quasi‐experiments work by identifying natural or institutional processes that approximate random assignment. For example, economists studying the fetal origins hypothesis have employed unexpected policy rollouts, natural disasters, famines, or infectious disease shocks to estimate the long‐term effects of early‐life conditions on adult health and socioeconomic outcomes (Almond and Currie 2011). These studies often draw on administrative or vital registration datasets with large sample sizes and high statistical power, allowing for the detection of relatively small but policy‐relevant causal effects. While such data sources differ from the intensive, fieldwork‐based data more common in human biology, the rise of population registries and large‐scale household panels offers new opportunities for secondary analysis and data linkage (Rosinger and Ice 2019).

While they circumvent the need to measure and adjust for all confounders, quasi‐experimental methods rely on strong assumptions that may not always be met in practice. For example, mendelian randomization (MR), a form of instrumental variables analysis, leverages the random assortment of genes at conception as a natural experiment, aiming to bypass confounding and reverse causation (Bonilla et al. 2023; Lawlor et al. 2008). Although promising in theory, MR is highly sensitive to violations of its identifying assumptions; most notably, that the genetic variant influences the outcome only through the exposure of interest (the exclusion restriction), and is not associated with confounders of the exposure–outcome relationship. In practice, pleiotropy (where genetic variants affect multiple traits) and population stratification (where allele frequencies vary across subpopulations) can bias results (Davey Smith and Hemani 2014). The recent proliferation of MR studies, particularly those using genome‐wide association study (GWAS) summary data, has prompted growing concern about false positives, reproducibility failures, and insufficient sensitivity analyses (Stender et al. 2024). For human biologists seeking to adopt MR or other quasi‐experimental methods, careful attention to study design, assumptions, and diagnostics is critical to ensure that quasi‐experimental inferences are both credible and meaningful.

A compelling illustration of realized causal inference using quasi‐experimental analysis and archival anthropological data comes from economic historians Feir et al. (2024), who examine the long‐term consequences of the near‐extinction of the North American bison for Indigenous nations historically reliant on them. The authors use a difference‐in‐differences design, a method that compares changes over time between groups affected and unaffected by a specific event, to estimate causal effects of the bison slaughter. Drawing on anthropometric data originally collected by Franz Boas (Boas 1895; Jantz 1995; Szathmáry 1995), they compare age‐adjusted height trends across birth cohorts in bison‐reliant and non‐reliant nations, finding a 2 to 3 cm decline in adult stature among bison nations, which erased their prior height advantage (Feir et al. 2024). The authors also use historical administrative and census data to show that the loss of the bison led to a 16 percentage point increase in child mortality, skewed sex ratios indicative of maternal deprivation, and a 19 percentage point decrease in the likelihood of working‐age men reporting an occupation. Finally, using contemporary data from the American Community Survey, they demonstrate that per capita income has remained approximately 25% lower among bison nations through the early 21st century. By tracing these intergenerational effects through a historically specific ecological collapse, the study demonstrates how quasi‐experimental methods and archival data can support realized causal inference by linking ecological and social disruptions to long‐term biocultural outcomes.

By becoming conversant with quasi‐experimental methods, human biologists can more effectively engage, critique, and contribute to this growing literature and expand our causal toolkit for understanding the biocultural production of human growth, health, and development.

### 3.3 Optimize Transparency and Reproducibility Where Possible

Our toolkit comes at a time when there has been an ongoing scientific crisis concerning analysis reproducibility, publication bias, and ethical violations in research practices that others have discussed at length (Ioannidis 2005; Munafò et al. 2017). Here, we emphasize that our job as researchers is not to tell an exciting story, but to tell a truthful one, grounded in sound research design and transparent communication. This is something that we can all work to improve, regardless of career stage. Thus, we suggest researchers at all stages to engage with and adopt practices of research transparency and reproducibility, where possible.

It is important to note the distinction between reproducibility and replicability. Reproducibility refers to the ability to obtain consistent results using the same data and analytical approach, and it is particularly critical in human biology, where studies are often highly contextual and challenging to replicate. Replicability, on the other hand, is more relevant in well‐defined experimental settings, such as clinical trials or psychology experiments, where researchers can closely emulate the study design and conditions. While replicability remains important for fields that can precisely recreate experimental conditions, reproducibility of analytical pipelines within studies is a greater concern in human biology.

The causal inference roadmap itself is an opportunity for enhancing transparency and reproducibility. At every stage from formulating a research question to analyzing the data, the roadmap provides a chance to clarify decisions, assumptions, and methodological choices. If exploratory or confirmative analyses have not begun, online preregistration of an analysis plan that is structured by the causal inference roadmap offers a powerful tool for enhancing transparency and reproducibility of the analytic approach.

Beyond preregistration, researchers can enhance transparency and replicability by sharing their data and analysis code when possible. In cases where data sharing may pose ethical concerns, software can be used to create an anonymized synthetic dataset that mimics key patterns in the data (Nowok et al. 2016; Quintana 2020).

We understand that many researchers are not able to share their data for complex reasons; however, do not let perfect be the enemy of good. Begin with smaller steps, even if it is a simple DAG, a preregistered analysis plan, or the sharing of analysis code. Even if true replication of observational studies in human biology may not always be feasible due to contextual factors, maximizing reproducibility and transparency wherever possible can meaningfully enhance the robustness of our field.

### 3.4 Toward Anthropological Causal Inference: Weaving Together Theory, Fieldwork, and Multiple Methods

The causal inference roadmap we have outlined is not an endpoint, but a scaffold for a broader project: building an anthropological approach to causal inference that is both rigorous and epistemologically grounded. Rather than seeking universal, interventionist effect estimates alone, human biologists aim to explain variation in health and biology through ecologically and culturally situated processes. Realized causal inference takes up this task by retrospectively explaining how causes unfolded in context, rather than simply imagining how an intervention might change outcomes under ideal conditions. In this tradition, inference to the best explanation (IBE) becomes essential: assessing the plausibility of causal claims by drawing on diverse forms of evidence, including long‐term fieldwork, ethnographic insight, and biological data. The biocultural synthesis provides a foundation for this work, emphasizing that biological outcomes are shaped not just by proximate exposures, but by lived experiences and structural conditions that accumulate across the life course (Fuentes 2023; Glass and Emmott 2025; Goodman and Leatherman 1998; Leatherman and Hoke 2017). By weaving together theoretical frameworks, fieldwork‐based understanding, and multiple methodological approaches, human biologists can develop causal explanations that are not only statistically credible, but anthropologically meaningful.

Recent works in evolutionary human behavioral ecology provide illuminating examples of how ethnographic research can reveal causal pathways that are often overlooked by quantitative models. For example, Brandl and Colleran (2024) illustrate how ethnographic research on bride price in Melanesia reveals multiple causal pathways that may or may not harm women, illustrating how nuanced, context‐specific factors shape causality beyond singular exposure–outcome relationships. Similarly, Snodgrass et al. (2024) explore ethnographic approaches to causal inference in their work on games, avatar identities, and play. Similar to the philosophy of inference to the best explanation, they discuss “abductive” logic in ethnography (drawing conclusions from observation and cumulative knowledge) alongside “deductive” and “inductive” approaches, underscoring how cultural, social, and behavioral dimensions enrich understanding of causation beyond what traditional statistical methods reveal.

By *triangulating* theory and fieldwork with quantitative and qualitative evidence, human biologists can contribute to causal explanations that include and extend beyond the narrow focus of estimating causal effects of exposures on outcomes (Lawlor et al. 2016; Morse 1991). We encourage researchers to think through how disparate forms of evidence inform each other: *Are they congruent or discordant? Do they mount evidence for or against your causal claim? What does one source potentially reveal*, and *what does another potentially conceal?* This includes not only qualitative and ethnographic data, but also the comparison of results across different quantitative designs, each with distinct sources of bias (Munafò and Davey Smith 2018). For instance, agreement between conclusions across studies with plausibly different magnitudes or sources of selection bias or unmeasured confounding can strengthen confidence in a causal interpretation. Considering diverse forms of evidence enables human biologists to deepen causal insights, especially given our frequent work in varied contexts such as field sites, archival datasets, or interdisciplinary teams.

## 4 Conclusion

Our roadmap for causal inference offers a structured approach for human biologists to enhance the rigor and transparency of their research. By focusing on the fundamental steps of clearly defining a causal research question, carefully identifying confounders using DAGs, and assessing causal assumptions before engaging in statistical estimation, human biologists can continue to clarify and refine our understandings of the complex relationships between biology, ecology, and culture. While challenges in observational research remain for the estimation of causal effects, integrating causal inference principles along with the strengths of observational anthropological fieldwork and theoretical grounding can push the field forward by fostering research that is both scientifically rigorous and deeply contextualized within human experiences.

## Author Contributions

**Elijah J. Watson:** conceptualization (equal); writing – original draft preparation (equal); writing – review and editing (equal). **Delaney J. Glass:** conceptualization (equal); writing – original draft preparation (equal); writing – review and editing (equal). **Lucia C. Petito:** writing – original draft preparation (supporting); writing – review and editing (equal).

## Ethics Statement

The authors have nothing to report.

## Conflicts of Interest

The authors declare no conflicts of interest.

## Data Availability

Data sharing is not applicable to this article as no new data were created or analyzed in this study.

## Endnotes

^1^
Causal inference with continuous exposures ideally involves estimating a dose–response curve (Zhang et al. 2016). The coefficient from a traditional linear model assumes a constant effect of a continuous exposure across its full range, which is a strong causal assumption about how the exposure operates. As such, causal interpretations of linear models with continuous exposures should be made cautiously. More flexible modeling strategies can better capture complex dose–response relationships, but they introduce additional assumptions and analytic degrees of freedom that must be transparently conveyed. Still, even when linear models are used with continuous exposures, as is common in human biology, the core principles of the causal roadmap remain applicable, since the causal assumptions regarding exchangeability, positivity, and exposure construct validity still require careful attention.
